# Chronic Disease, Disability, Psychological Distress and Suicide Ideation among Rural Elderly: Results from a Population Survey in Shandong

**DOI:** 10.3390/ijerph15081604

**Published:** 2018-07-28

**Authors:** Jing Zhu, Lingzhong Xu, Long Sun, Jiajia Li, Wenzhe Qin, Gan Ding, Qian Wang, Jiao Zhang, Su Xie, Zihang Yu

**Affiliations:** 1 Key Lab of Health Economics and Policy Research, School of Public Health, Shandong University, 44 Wen-hua-xi Road, Jinan 250012, China; zj1176559133@163.com (J.Z.); sunlong@sdu.edu.cn (L.S.); lijiajia@sdu.edu.cn (J.L.); qinwenzhe09@163.com (W.Q.); dinggan90@163.com (G.D.); wangqian0519@126.com (Q.W.); 201614324@mail.sdu.edu.cn (J.Z.); xs340823@163.com (S.X.); 13249220350@163.com (Z.Y.); 2 Collaborative Innovation Center of Social Risks Governance in Health, School of Public Health, Fudan University, Shanghai 200032, China

**Keywords:** suicide ideation, psychological distress, chronic disease, ADL, rural elderly, path analysis

## Abstract

*Objective:* Suicide is a major public health and social problem in contemporary societies. Previous studies showed that the older the seniors were, the more likely it was that they would experience disability, chronic disease, or both. The objective of this study was to examine the joint effects of chronic disease and physical disability on suicide ideation while controlling for psychological distress among the rural elderly living in Shandong Province, China. *Method*: A total of 5514 rural elderly individuals (60+) living in Shandong Province, China were included in this study. Suicidal ideation was assessed by using questions from the National Comorbidity Survey (NCS). Multiple logistic analyses were performed to examine the factors associated with suicide ideation. A path analysis was conducted to test the direct and indirect effects of chronic disease and of activity of daily living (ADL) limitation on suicide ideation while controlling for psychological distress. *Results*: The prevalence of suicide ideation among the rural elderly in Shandong, China was 11.0%. Psychological distress had the strongest direct (β = 0.392) and total effect (β = 0.392), chronic disease (β = −0.034; β = −0.063) had both direct and indirect impacts, and ADL (β = 0.091) had indirect impacts on suicide ideation. Psychological distress was a mediator between chronic disease, ADL limitation, and suicide ideation. *Conclusions*: Psychological distress was the greatest influencing factor of suicide ideation among the rural elderly, followed by chronic disease and disability. Effective intervention measures should be taken to facilitate the early detection of psychological distress in clinical practice among the rural elderly.

## 1. Introduction

Suicide is a major public health and social problem in contemporary societies, and lays a huge economic, social, and psychological burden on individuals, families, communities, and countries. Every year, around 800,000 people worldwide die due to suicide, which corresponds to an adjusted global rate of 11.4/100,000 in 2012 [1]. China, where suicide is ranked as the fifth leading cause of death, accounts for an estimated 22% of global suicides, or roughly 200,000 deaths per year [2].

Although the suicide rates in China have decreased in the past two decades [3], the peak for the elderly has remained relatively unchanged and has even slightly increased in recent years, making suicide among the elderly in China a prevalent public health problem which we should address [4]. Some previous studies conducted in China and other countries have indicated that the suicide rate was higher among the elderly [5,6]. In addition, the rural elderly suicide rates are three to five times higher than those in urban areas [7,8]. Suicide ideation is an important risk factor for attempted and completed suicide [9]. For suicide ideation, S. H. Kim found that chronic illnesses and functional limitations were associated with an increased risk of suicide ideation [10]. H. Xu, L. Qin, and J. Wang et al. had similar results [11]. Chronic disease and disability were also determinants of “high” psychological distress [12,13]. Social demographics and other factors such as depression and insomnia have been associated with suicide ideation [14,15,16].

Previous studies have identified the relationship between chronic disease, disability, psychological distress, and suicide ideation. However, there have been few ideas concerning the direct and indirect effects of activity of daily living (ADL) limitation, chronic disease, and psychological distress on suicide ideation. The purpose of the current study is to examine the joint effects of chronic disease and physical disability on suicide ideation among the elderly while controlling for psychological distress. Specifically, chronic disease has a direct effect on suicide ideation, and both chronic disease and physical disability have an indirect effect on suicide ideation through psychological distress. In particular, the current study will address the following research questions and hypotheses. First, chronic disease and physical disability are associated with suicide ideation. Second, chronic disease has a partial direct effect and a partial indirect effect on suicide ideation through psychological distress. Third, physical disability has a full indirect effect on suicide ideation through psychological distress.

## 2. Method

### 2.1. Settings and Participants

**Figure 1:**
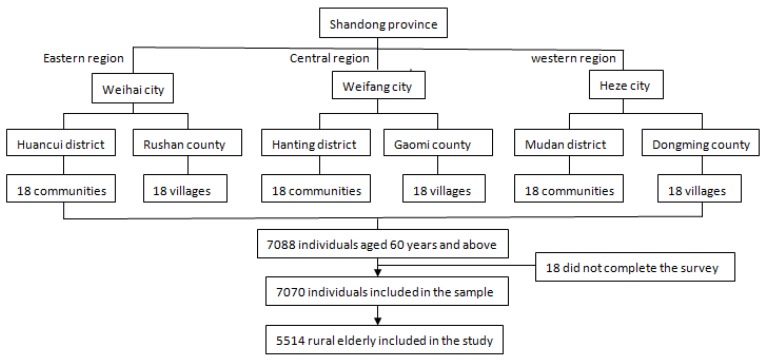
Flow chart of participant enrolment.

The design of the study consisted of face-to-face interviews using a structured questionnaire. This study was collected from the 2017 Survey of the Shandong Elderly Family Health Service, which was conducted by Shandong University. Stratified multi-stage random sampling was applied: in the first stage, six counties were selected from 137 counties as the primary sampling units (PSUs) throughout the eastern, central, and western regions of Shandong Province (which were divided into three districts and three counties that represented urban and rural areas, respectively). From each PSU, 18 villages in rural areas and 18 communities in suburban and urban areas were selected in the second stage as the secondary sampling units (SSUs). In the third stage, based on the roster of the residents by age and the total elderly population of each selected site provided by the local residential committee, an average of 66 individuals were stratified and randomly selected from each SSU, making up the total sample. The eligible participants for this survey were those aged 60 and above with local household registrations at the time of the interview. Initially, 7088 elderly individuals were selected and interviewed. Of these, 18 did not complete the survey because of an uncompleted questionnaire or an obvious logical error in the questionnaire. Finally, a total of 7070 individuals were included in the sample (Figure 1). Our current study focused on respondents who answered the question “Have you ever seriously thought about committing suicide?” among the rural elderly (*n* = 5514).

### 2.2. Variables and Measures

#### 2.2.1. Dependent Variable

Suicide ideation was assessed by using a question from the baseline National Comorbidity Survey (NCS) [17]: “Have you ever seriously thought about committing suicide?” The question was designed to ask whether they had experienced this idea in their lifetime. For the purpose of the analysis, a positive response was considered to indicate the presence of suicide ideation.

#### 2.2.2. Independent Variables

##### Physical Disability

The elderly individual’s physical disability was measured using the ADL Scale, including ADL subscales [18] and instrumental activity of daily living (IADL) subscales [19]. The ADL subscale assesses six types of ability: feeding, dressing, bathing, toileting, grooming, and locomotion. The IADL subscale evaluates the ability to perform eight types of more complex activities, like using a telephone, using transportation, and shopping. Scores for performing activities range from 1 to 4 (1 point for each activity performed without help and 4 points for each activity that the individual is unable to perform). The maximum score is 56 (higher scores indicate greater dependence). Edwards confirmed the ADL scales in a Brazilian study, showing a Cronbach’s α of 0.96–0.99 [20]. The Cronbach’s α for the ADL was 0.93 in this sample. 

##### Psychological Distress

Kessler 10 (K10) is a recognized and validated measure of psychological distress, in which respondents report how frequently they experienced specified symptoms of psychological distress [21,22]. An individual’s sense of psychological distress is evaluated against ten items on a self-report questionnaire. Each item is rated on a five-point scale with numerical values assigned to the responses (none of the time, a little of the time, some of the time, most of the time, and all the time). The ratings are summed to yield a total score, with higher scores indicating higher levels of psychological distress. The scale’s internal consistency, as assessed by Cronbach’s α, was 0.87. The Cronbach’s α for the K10 was 0.92 in this sample.

##### Socio-Demographic Characteristics

The socio-demographic characteristics included chronic diseases in the past 6 months, gender, age, education level, residential status, self-rated health, and family relationship. Chronic disease was coded as yes and no in the past 6 months. Gender was coded as male and female. Education level was coded as illiteracy, primary, and middle or above. Residential status was coded as single and couple. Self-rated health was coded as good and bad. Family relationship was coded as good and bad.

### 2.3. Statistical Analysis

All statistical analyses were performed using SPSS 22.0 (IBM Corp, Armonk, NY, USA) and Amos 21.0 (IBM Corp, Armonk, NY, USA). *t*-test or χ^2^ test was used to compare the difference in continuous or categorical variables. Logistic regression analysis was performed to examine the association of suicide ideation with some variables. Gender, ADL, chronic disease, and psychological distress were chosen as the independent variables in a path analysis model. The reported CI was calculated at the 95% level, and statistical significance was set at the 5% level.

## 3. Results

### 3.1. Socio-Demographic Characteristics of the Sample

Table 1 displays the socio-demographic details of the rural elderly about suicide ideation. A total of 5514 rural elderly individuals responded to the study. About 11.0% (*n* = 605) acknowledged suicide ideation (see Table 1). The age of the participants ranged from 60 to 101 (mean= 69.72; SD = 6.52), 57.1% were female, 80.8% lived with a spouse, and 39.2% was illiterate. Of the participants, 94.6% had a harmonious family. Furthermore, those people who had chronic disease accounted for 68.8%, whose self-rated health status was good accounted for 52.2%. The study compared participants in terms of the variables of age, gender, education level, residential status, self-rated health, family relationship, and chronic disease status in relation to suicide ideation. The participants differed significantly from each other in relation to suicide ideation.

### 3.2. Logistic Regression Analysis

The results of the logistic regression analysis of suicide ideation are shown in Table 2. ADL and chronic disease were significantly associated with suicide ideation (Models 1 and 2). When we adjusted for psychological distress, the OR of ADL changed from 1.03 to 0.99 (Models 1 and 3) and the chronic disease status changed from 1.71 to 1.14 (Models 2 and 4). Age (OR = 0.97), education (primary OR = 0.73, middle or above OR = 0.49) and family relationship (OR = 1.39) were significantly associated with suicide ideation among the rural elderly in Shandong, China (Model 5).

### 3.3. The Mediating Effect of Psychological Distress between ADL Limitation and Suicide Ideation

**Figure 2:**
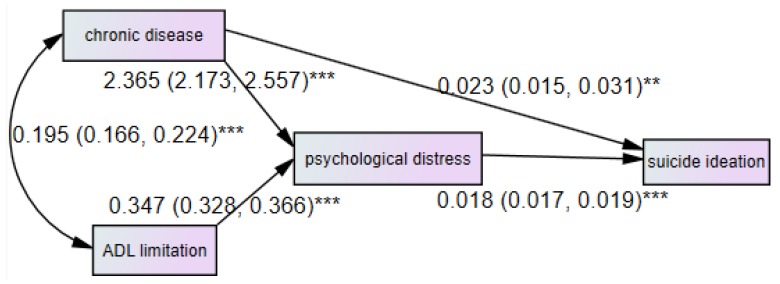
Direct and indirect effects of chronic disease, disability, and psychological distress on suicide ideation among the rural elderly in Shandong, China (OR and its 95% CI). ** *p* < 0.01, *** *p* < 0.001.

Figure 2 demonstrates the interaction between ADL limitation, chronic disease, psychological distress, and suicide ideation. Standardized regression coefficients are presented on each arrow. Using a path analysis, we found that psychological distress had a direct impact on suicide ideation. The influences of ADL on suicide ideation were mediated by psychological distress. Chronic disease had both direct and indirect effects on suicide ideation. The model had an absolute fit index (GFI) of 1.000, a simple fit index (P) of 0.866, a comparative fit index (CFI) of 1.000, and a root mean square error of approximation (RMSEA) of 0.000. Four fit indices indicated that the final path model adequately represented the data.

Table 3 presents the total, direct, and indirect effects of the variables on suicidal ideation. The total effects are the sum of the direct and indirect effects. Indirect effects represent the effects of one variable on another variable through indirect routes. We found that the value of standardized total effects of psychological distress was (0.392), while chronic disease was 0.096 and ADL limitation was 0.091.

## 4. Discussion

The lifetime prevalence of suicide ideation among the rural elderly was 11.0% (605 of 5514) in the current study. It was lower than the lifetime prevalence of 13.0% among the rural elderly living in Hunan Province, China [23]. It was higher than the reported rates among the elderly in Shandong, China (4.2%) [24], Hong Kong (5.5%) [25], Taiwan (6.1%) [26], and Canada (8.4%) [27]. Because of the different levels of research backgrounds and the time point of definition, the prevalence of suicide ideation varied widely.

Psychological distress had the most powerful and direct relationship with suicide ideation in the current study. This is similar to previous studies. For example, one study found that psychological distress had a direct and mediating role in suicidal patients among adolescents [28], which was also a predictor for suicide ideation among the community-dwelling elderly [29]. This correlation was stronger in older people than in younger people [27]. The K10 scale was used to represent psychological distress. Psychological distress was an effective screener to measure suicide ideation, which was composed of two primary factors (depression and anxiety) [30,31]. 

In the current study, when psychological conditions were controlled, ADL limitation had little direct effect on suicide ideation. But suicide ideation was indirectly accompanied by psychological distress, which implies that psychological distress mediates the process of developing suicide ideation. Studies demonstrated that disability was significantly associated with suicidal ideation, particularly for the elderly [32,33]. Functional disability as a determinant of “high” psychological distress has been demonstrated by several studies [34]. One possible explanation for the mediating role of psychological distress was the lack of their daily housework and economic resources.

This study shows that chronic disease had both direct and indirect impacts on suicide ideation. Studies have found a positive association between chronic disease and suicide, such as coronary heart disease, diabetes and combined illness, etc. [35,36,37], which was similar to the findings of our study. Using a path analysis, we proposed that chronic disease would affect suicide ideation directly or through psychological distress, and we found that it had both direct and indirect effects on suicide ideation among the elderly. Some previous studies demonstrated that chronic disease was associated with psychological distress among the elderly [38,39,40]. Furthermore, psychological distress was found to be a predictor for suicide ideation among the elderly [41]. One possible explanation was that high levels of disease burden were more sensitive to psychological distress. In addition, the internet might also have positive effects on particularly older people who obtain information about their (chronic) diseases that contribute to their stress and access social support that is beneficial to their mental health [42,43,44]. There is a considerable body of research on the effects, both positive and negative, of media on suicide among different age groups [45,46,47,48,49]. The implication of this finding was that chronic disease may not only directly affect, but also prevent suicide ideation by reducing the psychological distress among elderly people.

Age, education, and family relationship were significantly associated with suicide ideation in our study, but we mainly studied the relationship between chronic disease, ADL limitation, psychological distress, and suicide ideation.

### Strength and Limitations

The large sample size (*n* > 5000) provided strong statistical support for our study. However, the study still has several limitations, as indicated below. First, this study was based on a house-to-house interview, but village clinic interviews and house-to-house interviews were combined. The results may be biased because the individuals who arrived to the clinics usually had normal ADL, whereas the elderly with ADL limitation may not have been able to be interviewed. Second, information, including suicide ideation and health status, was self-reported, inevitably leading to the possibility of subjective bias. Third, we mainly focused on the risk factors for suicide ideation in the current study, and gave little consideration to the relationship between suicide ideation and attempted and completed suicide. Fourth, the dependent variable was lifetime suicide ideation. Chronic disease was diagnosed clearly by the medical staff in the first half of the investigation, but this study could not determine if suicide ideation occurred before, after, or both before and after experiencing psychological distress and health problems. Finally, the ORs for all of these variables were low. There were many statistically significant findings, but the effects noted in the data provided a limited ability to predict suicide ideation.

## 5. Conclusions

Psychological distress was found to have the greatest total and direct effect on suicide ideation among rural elderly, followed by chronic disease and disability. Chronic disease had a partial direct effect and a partial indirect effect on suicide ideation through psychological distress, but disability only had an indirect effect on suicide ideation through psychological distress. These findings imply a need to take effective intervention measures to facilitate early detection of psychological distress in clinical practice among the rural elderly.

## Figures and Tables

**Table 1:** Socio-demographic factors associated with suicide ideation among the rural elderly in Shandong, China.

Variable	*N* (%)	Suicide Ideation			
		No (%)	Yes (%)	χ^2^/t	*p*-Value
Observations	5514	4909 (89.0)	605 (11.0)		
Age	69.72 ± 6.52	68.81 ± 6.18	69.83 ± 6.55	5.732	0.000
Gender				26.025	0.000
Male	2366 (42.9)	2165 (91.5)	201 (8.5)		
Female	3148 (57.1)	2744 (87.2)	404 (12.8)		
Education				33.483	0.000
Illiteracy	2164 (39.2)	1868 (86.3)	296 (13.7)		
Primary	2277 (41.3)	2045 (89.8)	232 (10.2)		
Middle or above	1073 (19.5)	996 (92.8)	77 (7.2)		
Residential status				7.292	0.009
Single	1060 (19.2)	919 (86.7)	141 (13.3)		
Couple	4454 (80.8)	3990 (89.6)	464 (10.4)		
Self-rated health				110.342	0.000
Good	2878 (52.2)	2684 (93.3)	194 (6.7)		
Bad	2636 (47.8)	2225 (84.4)	411 (15.6)		
Family relationship				129.485	0.000
Good	5218 (94.6)	4705 (90.2)	513 (6.7)		
Bad	296 (5.4)	204 (84.4)	92 (15.6)		
Chronic disease status				60.350	0.000
No	1718 (31.2)	1613 (93.9)	105 (6.1)		
Yes	3796 (68.8)	3296 (86.8)	500 (13.2)		

**Table 2:** Logistic regression analysis for predictors of multiple suicide ideation (OR and its 95% CI).

Variables	Model 1	Model 2	Model 3	Model 4	Model 5
Age	0.96 (0.94–0.97) ***	0.96 (0.95–0.98) ***	0.97 (0.95–0.98) ***	0.97 (0.95–0.98) ***	0.97 (0.95–0.98) ***
Gender (Male)					
Female	1.29 (1.06–1.57) *	1.25 (1.03–1.53) *	1.10 (0.89–1.36)	1.08 (0.87–1.33)	1.08 (0.87–1.33)
Education (Illiteracy)					
Primary	0.81 (0.67–0.99) *	0.80 (0.66–0.97) *	0.73 (0.59–0.90) *	0.74 (0.60–0.91) **	0.73 (0.59–0.90) **
Middle or above	0.58 (0.43–0.77) ***	0.57 (0.43–0.76) ***	0.49 (0.36–0.67) ***	0.49 (0.36–0.67) ***	0.49 (0.36–0.66) ***
Residential status (single)					
Couple	0.74 (0.60–0.92) **	0.76 (0.61–0.94) *	0.80 (0.63–1.01)	0.80 (0.64–1.02)	0.80 (0.64–1.02)
Self-rated health (Good)					
Bad	2.20 (1.83–2.65) ***	3.39 (2.58–4.45) ***	1.29 (1.05–1.59) *	1.17 (0.95–1.44)	1.18 (0.96–1.46)
Family relationship (Good)					
Bad	3.12 (2.36–4.12) ***	2.01 (1.66–2.44) ***	1.38 (1.01–1.90) *	1.37 (1.00–1.88) *	1.39 (1.01–1.91) *
Chronic disease (No)					
Yes		1.71 (1.36–2.16) ***		1.14 (1.13–1.16) ***	1.14 (1.13–1.16) ***
ADL	1.03 (1.01–1.04) ***		0.99 (0.98–1.01)		0.99 (0.98–1.01)
Psychological distress			1.14 (1.13–1.16) ***	1.44 (1.13–1.85) **	1.44 (1.13–1.85) **

ADL: activity of daily living, * *p* < 0.05, ** *p* < 0.01, *** *p* < 0.001.

**Table 3:** Standardized effects on suicide ideation from path analysis on psychological distress, chronic disease and activity of daily living (ADL) among the rural elderly in Shandong, China.

	Total Effect	Direct Effect	Indirect Effect
Chronic disease	0.096	0.034	0.063
ADL	0.091	0.000	0.091
Psychological distress	0.392	0.392	0.000

## References

[1] WHO (2014). Preventing Suicide: A Global Imperative. http://www.who.int/entity/mental_health/suicide-prevention/exe_summary_english.pdf?ua=1.

[2] Cui W. (2009). Women and suicide in rural China. Bull. World Health Organ..

[3] Zhang J., Ma J., Jia C., Sun J., Guo X., Xu A., Li W. (2010). Economic growth and suicide rate changes: A case in China from 1982 to 2005. Eur. Psychiatry.

[4] Wang C.W., Chan C.L.W., Yip P.S.F. (2014). Suicide rates in China from 2002 to 2011: An update. Soc. Psychiatry Psychiatr. Epidemiol..

[5] Terranova C., Cardin F., Bruttocao A., Militello C. (2012). Analysis of suicide in the elderly in Italy. Risk factors and prevention of suicidal behavior. Aging Clin. Exp. Res..

[6] Li X., Xiao Z., Xiao S. (2009). Suicide among the elderly in mainland China. Psychogeriatrics.

[7] Phillips M.R., Li X., Zhang Y. (2002). Suicide rates in China. Lancet.

[8] Yip P.S., Liu K.Y., Hu J., Song X.M. (2005). Suicide rates in China during a decade of rapid social changes. Soc. Psychiatry Psychiatr. Epidemiol..

[9] Goldstein R.B., Black D.W., Nasrallah A., Winokur G. (1991). The prediction of suicide, Sensitivity, specificity, and predictive value of a multivariate model applied to suicide among 1906 patients with affective disorders. Arch. Gen. Psychiatry.

[10] Kim S.H. (2016). Suicidal ideation and suicide attempts in older adults: Influences of chronic illness, functional limitations, and pain. Geriatr. Nurs..

[11] Xu H., Qin L., Wang J., Zhou L., Luo D., Hu M., Li Z., Xiao S. (2016). A cross-sectional study on risk factors and their interactions with suicidal ideation among the elderly in rural communities of Hunan, China. BMJ Open.

[12] Korda R.J., Paige E., Yiengprugsawan V., Latz I., Friel S. (2014). Income-related inequalities in chronic conditions, physical functioning and psychological distress among older people in Australia: Cross-sectional findings from the 45 and up study. BMC Public Health.

[13] Sampasa-Kanyinga H., Zamorski M.A., Colman I. (2018). Mental Disorder, Psychological Distress, and Functional Status in Canadian Military Personnel. Can. J. Psychiatry.

[14] Yen Y.C., Yang M.J., Yang M.S., Lung F.W., Shih C.H., Hahn C.Y., Lo H.Y. (2005). Suicidal ideation and associated factors among community-dwelling elders in Taiwan. Psychiatry Clin. Neurosci..

[15] Kang H.J., Stewart R., Jeong B.O., Kim S.Y., Bae K.Y., Kim S.W., Kim J., Shin I., Yoon J.S. (2014). Suicidal ideation in elderly Korean population: A two-year longitudinal study. Int. Psychogeriatr..

[16] Pompili M., Innamorati M., Forte A., Longo L., Mazzetta C., Erbuto D., Palermo F.R., Stefani H., Seretti M.E., Lamis D.A. (2013). Insomnia as a predictor of high-lethality suicide attempts. Int. J. Clin. Pract..

[17] Lipschitz D.S., Winegar R.K., Nicolaou A.L., Hartnick E., Wolfson M., Southwick S.M. (1999). Perceived abuse and neglect as risk factors for suicidal behavior in adolescent inpatients. J. Nerv. Ment. Dis..

[18] Katz S. (1963). Studies of illness in the aged. The index of ADL: A standardized measure of biologic and psychologic function. JaMa.

[19] Lawton M.P., Brody E.M. (1969). Assessment of older people: Self-maintaining and instrumental activities of daily living. Gerontologist.

[20] Edwards M.M. (1990). The reliability and validity of self-report activities of daily living scales. Can. J. Occup. Ther..

[21] Cairney J., Veldhuizen S., Wade T.J., Kurdyak P., Streiner D.L. (2007). Evaluation of 2 measures of psychological distress as screeners for depression in the general population. Can. J. Psychiatry.

[22] Kessler R.C., Andrews G., Colpe L.J., Hiripi E., Mroczek D.K., Normand S.L., Walters E.E., Zaslavsky A.M. (2002). Short screening scales to monitor population prevalences and trends in non-specific psychological distress. Psychol. Med..

[23] Tao R.Q., Zeng Z., Zhong G.L., Cha W.T., Liang W.J. (2011). A case-control study of suicidal ideation among rural elderly in a city in Hunan province. J. Hum. Norm. Univ. Med. Edit.

[24] Ge D., Sun L., Zhou C., Qian Y., Zhang L., Medina A. (2017). Exploring the risk factors of suicidal ideation among the seniors in Shandong, China: A path analysis. J. Affect. Disord..

[25] Yip P.S., Chi I., Chiu H., Chi Wai K., Conwell Y., Caine E. (2003). A prevalence study of suicide ideation among older adults in Hong Kong SAR. Int. J. Geriatr. Psychiatry.

[26] Chan H.L., Liu C.Y., Chau Y.L., Chang C.M. (2011). Prevalence and association of suicide ideation among Taiwanese elderly—A population-based cross-sectional study. Chang Gung Med. J..

[27] Vasiliadis H.M., Gagné S., Jozwiak N., Préville M. (2013). Gender differences in health service use for mental health reasons in community dwelling older adults with suicidal ideation. Int. Psychogeriatr..

[28] Puuskari V., Aalto-Setälä T., Komulainen E., Marttunen M. (2018). Suicidal ideation, suicide attempts, and psychological distress among intoxicated adolescents in the pediatric emergency department. Nord. J. Psychiatry.

[29] Fujita K., Yong R., Sasaki H., Kaneko Y., Eboshida A., Motohashi Y. (2016). Psychological distress as a predictor of suicide ideation among the community-dwelling elderly. Gerontologist.

[30] O’Connor S.S., Beebe T.J., Lineberry T.W., Jobes D.A., Conrad A.K. (2012). The association between the Kessler 10 and suicidality: A cross-sectional analysis. Compr. Psychiatry.

[31] Chamberlain P., Goldney R., Delfabbro P., Gill T., Dal Grande L. (2009). Suicidal ideation: The clinical utility of the K10. Crisis.

[32] Brooks R.T., Beard J., Steel Z. (2006). Factor structure and interpretation of the K10. Psychol. Assess..

[33] Dennis M., Baillon S., Brugha T., Lindesay J., Stewart R., Meltzer H. (2009). The influence of limitation in activity of daily living and physical health on suicidal ideation: Results from a population survey of Great Britain. Soc. Psychiatry Psychiatr. Epidemiol..

[34] Zhang L., Sun L., Zhou C., Ge D., Qian Y. (2018). The Relationship Between Difficulties in Daily Living and Suicidal Ideation Among Older Adults: Results from a Population-Based Survey in Shandong. J. Nerv. Ment. Dis..

[35] Štefan L., Sporiš G., Krističević T. (2018). Are lower levels of physical activity and self-rated fitness associated with higher levels of psychological distress in Croatian young adults? A cross-sectional study. PeerJ.

[36] Joshi P., Song H.B., Lee S.A. (2017). Association of chronic disease prevalence and quality of life with suicide-related ideation and suicide attempt among Korean adults. Indian J. Psychiatry.

[37] Conti C., Mennitto C., Di Francesco G., Fraticelli F., Vitacolonna E., Fulcheri M. (2017). Clinical characteristics of diabetes mellitus and suicide risk. Front. Psychiatry.

[38] Qin P., Hawton K., Mortensen P.B., Webb R. (2014). Combined effects of physical illness and comorbid psychiatric disorder on risk of suicide in a national population study. Br. J. Psychiatry.

[39] Ojike N., Sowers J.R., Seixas A., Ravenell J., Rodriguez-Figueroa G., Awadallah M., Zizi F., Jean-Louis G., Ogedegbe O., McFarlane S.I. (2016). Psychological distress and hypertension: Results from the National Health Interview Survey for 2004–2013. Cardiorenal Med..

[40] Li C., Liu J.C., Xiao X., Chen X., Yue S., Yu H., Tang N.J. (2017). Psychological distress and type 2 diabetes mellitus: A 4-year policemen cohort study in China. BMJ Open.

[41] Russ T.C., Kivimäki M., Morling J.R., Starr J.M., Stamatakis E., Batty G.D. (2015). Association between psychological distress and liver disease mortality: A meta-analysis of individual study participants. Gastroenterology.

[42] Abreu W., Tolson D., Jackson G.A., Costa N. (2018). A cross-sectional study of family caregiver burden and psychological distress linked to frailty and functional dependency of a relative with advanced dementia. Dementia.

[43] Arendt F., Scherr S. (2017). The impact of a highly publicized celebrity suicide on suicide-related online information seeking. Crisis.

[44] Haim M., Arendt F., Scherr S. (2017). Abyss or shelter? On the relevance of web search engines’ search results when people google for suicide. Health Commun..

[45] Scherr S., Reinemann C. (2016). First do no harm: Cross-sectional and longitudinal evidence for the impact of individual suicidality on the use of online health forums and support groups. Comput. Hum. Behav..

[46] Reinemann C., Scherr S. (2011). Der Werther-Defekt: Plädoyer für einen neuen Blick auf den Zusammenhang von suizidalem Verhalten und Medien. Publizistik.

[47] Scherr S. (2013). Medien und Suizide: Überblick über die kommunikationswissenschaftliche Forschung zum Werther-Effekt. Suizidprophylaxe.

[48] Scherr S. (2016). Depression—Medien—Suizid: Zur Empirischen Relevanz von Depressionen und Medien Für Die Suizidalität.

[49] Scherr S., Steinleitner A. (2015). Zwischen dem Werther-und Papageno-Effekt. Nervenarzt.

